# The NLRP3 Inflammasome Gene Is Overexpressed in Hidradenitis Suppurativa Lesions: A Preliminary Study on the Role of Pyroptosis in Disease Pathogenesis

**DOI:** 10.3390/cimb46030161

**Published:** 2024-03-16

**Authors:** Piotr K. Krajewski, Weronika Szukała, Jacek C. Szepietowski

**Affiliations:** 1 Department of Dermatology, Venereology and Allergology, Wroclaw Medical University, Chalubinskiego 1, 50-368 Wroclaw, Poland; piotr.krajewski@umw.edu.pl; 2 Doctoral School of Exact and Natural Sciences, Jagiellonian University, Lojasiewicza 11, 30-348 Krakow, Poland; 3 Faculty of Biochemistry, Biophysics and Biotechnology, Department of General Biochemistry, Jagiellonian University, Gronostajowa 7, 30-387 Krakow, Poland

**Keywords:** hidradenitis suppurativa, NLRP3 inflammasome, pyroptosis, P2X7R, pathogenesis

## Abstract

Hidradenitis suppurativa (HS) is a debilitating inflammatory skin disorder, and its pathogenesis remains incompletely understood. This study aimed to investigate the role of the P2X7 receptor (P2X7R) and NLRP3 inflammasome in HS pathogenesis. RNA sequencing and real-time PCR were performed to assess the gene expression levels of P2X7R and NLRP3 in the skin biopsies of HS patients and healthy controls (HC). The results of our study revealed a significantly increased expression of the NLRP3 gene in both the lesional and perilesional skin of HS patients compared to healthy controls. Moreover, the mRNA levels of NLRP3 were significantly higher in lesional skin compared to non-lesional skin in HS patients, indicating the spread of inflammation to adjacent tissues. In contrast, no significant differences in P2X7R gene expression were observed between the three groups. These findings suggest the involvement of NLRP3 inflammasomes in HS pathogenesis, while P2X7R may not play a significant role in the disease. This research sheds light on the complex inflammatory pathways in HS, highlighting the potential of NLRP3 as a therapeutic target. Understanding the molecular mechanisms underlying HS is crucial for the development of targeted treatment modalities for this debilitating condition.

## 1. Introduction

Hidradenitis suppurativa (HS) is an inflammatory disorder of the pilosebaceous unit predominantly affecting young adults [1]. The pathogenesis of HS is yet to be fully understood. The vicious cycle of local and systemic inflammation, with an overproduction of pro-inflammatory cytokines and lack of their inhibition, seems to be a hallmark of the disease [1,2]. Recent studies on the topic led to the description of the multiple possible pathogenetic pathways involved in the development of skin lesions, including both B- and T-cell contribution, dendritic cell activation, neutrophilic infiltration, adipokine dysregulation, and an increase in the activity of pro-inflammatory pathways [3,4,5,6,7]. Moreover, it has been established that pro-inflammatory lifestyles, including obesity and smoking, may lead to the development and exacerbation of the disease [1,2,7].

Our group recently described an increased expression of monocyte chemotactic protein-1-induced protein-1 (MCPIP1) in transcriptional and translational levels [8]. MCPIP1 is a protein that is involved in the suppression of inflammation and the maintenance of skin homeostasis, the lack of which leads to spontaneous cutaneous inflammation [9]. It has been reported to be induced by an overinduction of TNFα, IL-1β, and NFκB pathway activation, which also play a role in HS development [1,10]. Moreover, in the study by Manfredini et al. [11], the authors reported an increased protein expression of P2X7 receptor (P2X7R) and NLRP3 (NOD-, LRR- and pyrin domain-containing protein 3) in HS lesions [11]. The combined function of P2X7R and NLRP3 leads to the development and maturation of IL-1β, enhancing the inflammation and, as a result, activating MCPIP1 [9,10,11].

Our study aimed to perform RNA sequencing in the lesional and perilesional skin of the P2X7R and NLRP3 genes to confirm their possible implication in the pathogenesis of HS.

## 2. Materials and Methods

This research was carried out in compliance with the principles outlined in the Declaration of Helsinki and received approval from the Ethics Committee of Wroclaw Medical University (KB-520/2018 and KB-779/2022). Prior to their participation in the study, all patients provided their consent by signing appropriate documentation.

### 2.1. Patients

The investigation involved the analysis of skin biopsy specimens derived from 5 individuals (RNA sequencing) and 15 individuals (Real-Time PCR) affected with HS who had undergone surgical excision of skin lesions at the Department of Dermatology, Venereology, and Allergology of Wroclaw Medical University during the period from June to November 2022. A dermatologist with expertise in HS conducted comprehensive physical assessments of all patients. The demographic data, including sex, age, weight, height, alcohol intake, and smoking, as well as personal and family history of HS and acne, were collected. Moreover, HS-specific data consisting of the duration of the disease, age of first lesions, prior treatments, and surgeries were recorded. The severity of the disease was assessed with the Hurley staging system and the International Hidradenitis Suppurativa Severity Score Scale (IHS4) [12,13].

Furthermore, skin samples from six age- and gender-matched healthy controls (HC) were taken from patients who underwent surgical procedures for non-malignant skin conditions.

### 2.2. Biopsy

Before the procedure, the dermatologist responsible for conducting the biopsy demarcated the specific area. Following this, the individuals received an injection comprising an anesthetic (2% lidocaine) combined with adrenaline to mitigate potential pain and discomfort, as well as minimize bleeding. Subsequently, two punch biopsies, each measuring 5 mm in diameter, were extracted from the designated site. One biopsy was obtained from the actively inflamed lesion, while the second was acquired from adjacent healthy-looking skin situated in close proximity to the chosen lesion, with a minimum separation distance of 2 cm.

### 2.3. RNA Isolation

Each of the skin samples obtained was preserved by freezing them in RNAlater (Sigma, Saint Louis, MO, USA) and stored at a temperature of −80 °C. To isolate the total RNA from these samples, they were subjected to homogenization in fenozol (A&A Biotechnology, Gdansk, Polska) with the aid of a tissue homogenizer manufactured by OMNI International (Kennesaw, GA, USA).

### 2.4. RNA Sequencing

RNA sequencing was performed as a part of a bigger study by our team, which is currently under consideration for publication. The exact methodology has been described there. Functional Gene Ontology enrichment analysis of differentially expressed genes (DEGs) meeting the criteria of a fold change greater than 1.5 and an adjusted *p*-value less than 0.05 was conducted. This analysis was carried out using the R package ClusterProfiler version 4.4. The gene lists were studied utilizing Ensembl gene annotations (ENSEMBL_GENE_ID), with the background dataset derived from *Homo sapiens*. To visualize the results, volcano plots and dot plots were generated using the ggplot2 libraries in R. Venn diagrams were constructed using an online tool that can be accessed freely at (http://bioinformatics.psb.ugent.be/webtools/Venn accessed on 10 November 2023). Additionally, heatmaps were generated using GraphPad Prism 8. RNAseq data are available at GEO under accession no. GSE245451.

### 2.5. Quantitative Real-Time PCR

The quality and concentration of the total RNA were evaluated using a NanoDrop 1000 spectrophotometer from Thermo Fisher Scientific (Waltham, MA, USA). Subsequently, 1 μg of total RNA underwent reverse transcription using oligo(dT) primer and M-MLV reverse transcriptase from Promega (Madison, WI, USA). The resulting cDNA was then diluted five-fold, and real-time PCR assays were conducted employing the QuantStudio 3 system from Applied Biosystems, Thermo Fisher Scientific (Waltham, MA, USA), with SYBR Green qPCR master mix sourced from A&A Biotechnology (Gdynia, Poland).

The quantification of P2XR7 and NLRP3 transcript levels was normalized relative to elongation factor-2 (EF2) using the 2^−ΔCt^ method. The following gene-specific primer pairs were used: for EF2: GACATCACCAAGGGTGTGCAG and TTCAGCACACTGGCATAGAGGC; for NRLP3: TGAGCACCAGCCAGAGTCTA and GTGCTTCAGTCCCACACACA; for P2XR7: TCGTGGAGAATGGAGTGAAGAAG and TTCCGGTCTGAATTCCTTTGCT.

### 2.6. Statistical Analysis

The statistical analysis of the obtained results involved using IBM SPSS Statistics version 26 software, developed by SPSS Inc. in Chicago, IL, USA. We conducted an assessment of all data to determine whether they followed a parametric or non-parametric distribution. Subsequently, we computed the minimum, maximum, mean, and standard deviation values. We employed the Mann–Whitney U test, Spearman and Pearson correlations for the analysis of quantitative variables and used the Chi-square test for qualitative data. To compare mRNA and protein expression levels between two HS samples and healthy skin, we utilized one-way ANOVA. We considered a two-sided *p*-value of ≤0.05 to indicate statistical significance.

## 3. Results

### 3.1. Studied Group

#### 3.1.1. RT-PCR

The study cohort comprised fifteen individuals diagnosed with HS: seven of them were females (46.67%), and eight were males (53.55%). The average age of the participants was 35.8 years with a standard deviation of 11.2 years. Considering the mean body mass index (BMI) of 30.1 ± 6.31 kg/m^2^, the majority of the population was categorized as obese. Among the subjects, eight individuals (53.6%) were active smokers, with an average of 9.6 ± 6.1 pack years. Seven individuals (46.7%) reported experiencing juvenile acne during their adolescence, while only two (13.3%) had a family history of HS. A detailed description of the studied group has already been published [8].

#### 3.1.2. RNA-seq

The studied group consisted of three females and two males, constituting 60% and 40%, respectively. The average age of the patients was 35.2 ± 12.0 years, with a mean body mass index (BMI) of 31.8 ± 7.3, indicating obesity. The average duration of HS was determined to be 9.4 ± 5.9 years, with the mean age of the first lesion assessed as 25.8 ± 12.2 years. The majority of the subjects (60%) were active smokers, with an average consumption of 9.8 ± 1.8 pack years. Regarding the disease severity, the evaluation using the IHS4 yielded a mean score of 20.2 ± 8.4 points, indicative of severe disease. According to the Hurley staging system, three patients were categorized as Hurley level 2, while two were classified as Hurley level 3. All the patients were previously treated, and all of them received at least 3 months of therapy with antibiotics (tetracyclines or a combination of clindamycin and rifampicin). Two patients (40%) were treated with retinoids, and one (20%) received a short course of steroids. Only one patient had undergone previous surgery for HS.

### 3.2. RT-PCR and RNA-seq

**Figure 1:**
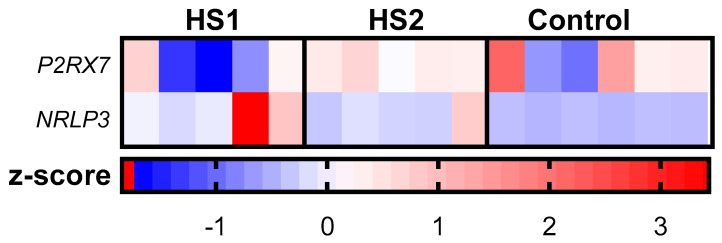
A heatmap depicting the z-score-based expression levels of differentially expressed genes (DEGs) identified through the Gene Ontology (GO) enrichment analysis for P2RX7 and NRLP3.

Total RNA was isolated from skin biopsies of the following nature: lesional skin of HS patients (HS-1); healthy-looking, non-lesional skin of HS patients (HS-2); and healthy skin from HC (HS = 5 patients; HC = 6 patients). We next employed mRNA sequencing (RNA-seq) to profile differences in the expression of P2RX7 and NRLP3 in HS patients’ skin based on the following three comparisons: HS-1 vs. HC, HS-2 vs. HC, and HS-1 vs. HS-2. The strongest difference was observed for the comparison of NRLP3 expression between HS1 and HC with a fold change of 19.39 and adjusted *p* < 0.0001. Moreover, a similar, yet three times weaker, association was visible in the comparison of HS2 with HC (fold change of 6.33, *p* < 0.001) (Table 1) (Figure 1).

Although there was a numerical difference recorded between HS1 and HS2, it did not achieve statistical significance. There was no difference in the expression of the P2RX7 gene between the three groups (Table 1) (Figure 1).

**Figure 2:**
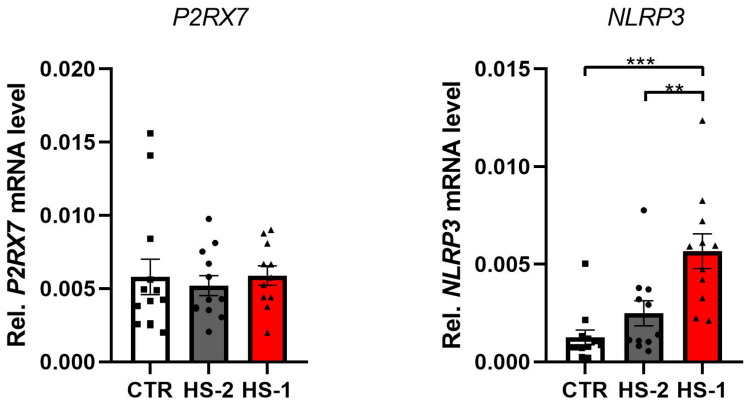
RT-PCR analysis for P2RX7 and NRLP3 genes in HS and control samples indicating relative levels of P2RX7 and NLRP3 mRNA in the skin samples taken from HS patients. ** *p* < 0.01, *** *p* < 0.001.

RT-PCR revealed statistically significant differences in relative mRNA levels of NLRP3 between the lesional (HS-1) and non-lesional (HS-2) skin of the affected patients (0.005665 ± 0.002485, respectively; *p* < 0.005) Moreover, statistically higher levels of NLRP3 mRNA were observed for HS-1 in comparison to HC (0.005665 ± 0.001253, respectively; *p* < 0.001) (Figure 2) (Table 2). A numerical yet statistically insignificant difference was observed for NLRP3 between HS-2 and HC. No differences were reported for relative mRNA levels of P2XR7 between the three studied groups (Figure 2) (Table 2).

## 4. Discussion

HS is a chronic, recurrent, inflammatory dermatosis primarily affecting young adults, with a peak incidence in patients between 20–40 years old [1]. The exact prevalence of the disease is unknown, yet according to the latest epidemiological studies, it may vary between 0.6–2.2% [14,15,16], with almost two-fold higher prevalence among women [17]. The disease is characterized by the appearance of recurrent, slowly healing, deeply seated inflammatory nodules or abscesses, which subsequently progress into purulent inflammatory tunnels and mutilating scarring [1,18]. HS has been widely related to a very high psychosocial burden for the patients and their families [19,20,21] and is frequently referred to as the worst dermatosis [22]. It has been associated with a higher prevalence and severity of psychiatric disorders, including anxiety and depression, as well as suicidal ideation [23,24,25]. Due to the high severity of associated pain, itch, and continuous purulent discharge [26], HS influences patients’ sleep quality [27], sexual life [28], satisfaction with life [29], and professional activity [30]. The management, including medical and procedural treatments, is highly unsatisfactory both for patients and physicians [31], with almost half of the patients dissatisfied with medical and/or procedural treatments [31]. Until recently, due to the lack of a full understanding of the pathogenesis, HS medical treatment has been mostly based on a general anti-inflammatory effect of drugs (i.e., antibiotics) rather than selected inflammatory pathways [32]. Multiple studies on the pathogenesis of HS and possible pathogenetic pathways revealed similarities with psoriasis, allowing the introduction of biologics, including FDA- and EMA-approved adalimumab and EMA-approved secukinumab [33,34]. Future research targeting possible therapeutic pathways is necessary for the introduction of HS-specific treatment modalities.

Nuclear factor-κB (NF-κB) constitutes a family of inducible transcription factors responsible for governing the expression of a wide spectrum of genes that play pivotal roles in various facets of immune and inflammatory responses [35]. The pathway may be activated in two ways: canonical and noncanonical (alternative). The canonical pathway responds to various stimuli, including growth factors, cytokines, microbial components, and stress agents [35,36], while the non-canonical pathway is primarily stimulated by a subset of TNF receptor (TNFR) superfamily members [35,36]. The NF-κB pathway serves as a central mediator for the activation of pro-inflammatory genes and operates within both innate and adaptive immune cell populations [35]. It is responsible for the induction of pro-inflammatory cytokines and chemokines and different types of mediators that act both directly through the induction of inflammation and indirectly through the promotion of the differentiation of inflammatory T-cells [35]. It has been documented that NF-κB plays a significant role in the development of multiple sclerosis, atherosclerosis, and rheumatoid arthritis, as well as inflammatory bowel diseases [35]. It is worth underlining that the NF-κB pathway is responsible for the activation and stimulation of multiple inflammatory pathways that are involved in the pathogenesis of HS, including the induction of IL-1, IL-6, TNFα, IL-23, and dendritic cell maturation, as well as T-cell activation and differentiation into Th1 and Th17 cells [3,4,5,6,7,35].

Moreover, NF-κB plays a pivotal role as the central mediator of the priming signal required for the activation of the NLRP3 inflammasome, which, according to the study by Lima et al. [37] is overexpressed in the lesional skin of HS [37]. The NLRP3 inflammasome is a protein involved in the release of inflammatory cytokines, as well as the induction of pyroptosis [38]. According to the authors, the NLRP3 inflammasome and its associated proteins are expressed in the epidermis of both the lesional and perilesional skin of HS patients [37]. Additionally, Western blot analysis demonstrated an increased expression of the active forms of NLRP3-associated proteins (i.e., caspase-1) in HS skin keratinocytes [37]. Moran et al. [39] described a significantly increased expression of NLRP3, CASP1, IL1B, IL18, IL23A, IL6, TGFB1, and TNF in HS patients in comparison to healthy controls [39]. Moreover, in an ex vivo study, the authors examined the influence of the inhibition of NLRP3, showing a significant decrease in IL-1β, TNF-α, IL-17A, IFN-γ, CCL20, CXCL1, IL-8, and IL-36γ, indicating a reduction in inflammatory response [39]. This is in accordance with the results of our study. We showed an increased expression of the NLRP3 gene in the lesional and perilesional skin of HS patients in comparison to healthy controls, confirming the function of NLRP3 in HS pathogenesis. Moreover, levels of mRNA for NLRP3 were significantly higher in the lesional skin of HS patients in comparison to non-lesional skin and HC. The increased expression of NLRP3 was also visible in the non-lesional, healthy-looking HS skin, indicating the spread of inflammation to adjacent tissues. In the study by Manfredini et al. [11], on the basis of the semi-quantitative evaluation of immunohistochemical (IHC) samples, the authors described the significantly increased expression of NLRP3 and P2X7R proteins in HS patients vs. HC [11]. There was also a weak positive association between the IHC expression of NLRP3 and P2X7R [11]. The authors proposed a codependent intracellular signaling pathway, which may be important to HS pathogenesis [11]. Unfortunately, the results of our study did not confirm these findings. Both gene expression and relative P2X7R mRNA levels did not differ between lesional and non-lesional skin. Moreover, no differences were found between HS patients and HC. The differences in the results may be attributed to the different methodological approaches. Manfredini et al. [11] performed a semi-quantitative IHC evaluation, while we performed detailed RNA-seq and RT-PCR. On the other hand, Manfredini et al. [11] analyzed the biopsies of 31 HS patients and 28 HC, while we used a total of 11 subjects (5 HS and 6 HC) for RNA-seq and 29 subjects for RT-PCR (14 HS and 15 HC). It is essential to underline that although P2X7R was previously studied in psoriasis and rosacea [40,41], it is not the sole method of NLRP3 activation [38]. It is thought that the activation of NLRP3 involves multiple, not mutually exclusive, upstream signals that encompass processes such as the efflux of potassium ions (K^+^) or chloride ions (Cl^−^), the influx of calcium ions (Ca^2+^), lysosomal disruption, mitochondrial dysfunction, metabolic alterations, and trans-Golgi disassembly [38]. Moreover, it was recently discovered that although the P2X family is non-selective regarding Na^+^, K^+^, and Ca^2+^ ion channels, P2X7 does not act as a channel for K^+^ efflux [42]. When activated, it instead mainly promotes Na^+^ and Ca^2+^ influx, mediating K^+^ efflux through the TWIK2 channel [38]. Interestingly, there is evidence indicating that Ca^2+^ influx may have an opposite function and leads to a decrease in NLRP3 and caspase-1 activation [43]. Therefore, the exact role of Ca^2+^ influx still needs to be elucidated.

We understand the limitations of our study. First, the study included only five HS patients and six HC for RNA-seq and only fifteen HS patients for RT-PCR. Nevertheless, according to the guidelines, six samples out of each group are sufficient for each condition [44]. Yet, future studies on bigger cohorts are necessary to confirm our findings. Second, this is a single-center study with only Caucasian patients; future studies with higher diversity may be required to generalize the observation to the general population. Lastly, we investigated only two genes in order to prove our hypothesis. We agree that studies with protein-level examination including complete transcriptome changes, such as immunohistochemistry or Western blotting, may be of benefit in better understanding HS pathogenesis.

## 5. Conclusions

In conclusion, the results of our study indicate the high expression of the NLRP3 gene in both the lesional and non-lesional skin of HS patients. This confirms previous findings and the role of NLRP3 inflammasomes in HS pathogenesis. Moreover, we have disproven the overexpression of P2X7R in HS patients, which indicates that NLRP3 in HS may be activated through different molecular pathways.

## Figures and Tables

**Table 1:** Differential expression results for P2X7R and NRLP3 genes based on sequencing.

Gene	HS-1 vs. HC		HS-2 vs. HC		HS-1 vs. HS-2	
	Fold Change	*p*	Fold Change	*p*	Fold Change	*p*
***P2X7R***	0.69	NS	1.19	NS	0.53	NS
***NRLP3***	19.39	**<0.0001**	6.33	**<0.001**	2.39	NS

HS-1—lesional skin of HS patients; HS-2—healthy-looking, non-lesional skin of HS patients; HC—healthy controls; NS—non-significant; P2X7R—P2X7 receptor; NRLP3—NOD-, LRR- and pyrin domain-containing protein 3; bold—statistically significant.

**Table 2:** Relative mRNA levels of NRLP3 and P2X7R assessed with RT-PCR.

Characteristic	HS-1	HS-2	HC	*p*	Post Hoc
NRLP3 (rel. mRNA level, mean ± SD)	0.005665 ± 0.00295421	0.002485 ± 0.002133171	0.001253 ± 0.001291779	<0.001	HS-1 vs. HS2: <0.005 HS-1 vs. HC <0.001 HS-2 vs. HC NS
P2X7R (rel. mRNA level, mean ± SD)	0.005885 ± 0.00220049	0.005201 ± 0.002356542	0.005802 ± 0.004355609	NS	NS

HS-1—lesional skin of HS patients; HS-2—healthy-looking, non-lesional skin of HS patients; HC—healthy controls; NS—non-significant; P2X7R—P2X7 receptor; NRLP3—NOD-, LRR- and pyrin domain-containing protein 3; rel.—relative; SD—standard deviation.

## Data Availability

Data are available from the corresponding author on a reasonable request.
